# Hemophagocytic Lymphohistiocytosis Secondary to Disseminated Histoplasmosis in Rheumatologic Disease

**DOI:** 10.1155/2021/6612710

**Published:** 2021-01-22

**Authors:** Yael Kusne, Michael Christiansen, Christopher Conley, Juan Gea-Banacloche, Ayan Sen

**Affiliations:** ^1^Department of Internal Medicine, Mayo Clinic Arizona, Phoenix, AZ, USA; ^2^Department of Neurology, Mayo Clinic Arizona, Phoenix, AZ, USA; ^3^Department of Pathology, Mayo Clinic Arizona, Phoenix, AZ, USA; ^4^Department of Infectious Disease, Mayo Clinic Arizona, Phoenix, AZ, USA; ^5^Department of Critical Care Medicine, Mayo Clinic Arizona, Phoenix, AZ, USA

## Abstract

**Background:**

Hemophagocytic lymphohistiocytosis (HLH) was originally described in pediatric patients presenting with fever, hepatosplenomegaly, and blood cell abnormalities. Later, HLH was recognized to occur in adults, often associated with hematologic malignancies or serious infections.

**Conclusion:**

Patients presenting with HLH are critically ill, and rapid diagnosis is key. In adults, the search for the trigger must begin promptly as time to diagnosis effects survival. The underlying trigger in our patients was Histoplasma capsulatum infection, which is rare in the southwestern United States. Prompt diagnosis led to recovery in one patient, while the other did not survive.

## 1. Background

Hemophagocytic lymphohistiocytosis (HLH) is a syndrome of immune overactivation that can lead to tissue destruction and organ failure. As a primary disorder, it is typically seen in pediatrics and has several associated genetic mutations [1]. In adults, it presents as a secondary phenomenon, seen in conjunction with infection, malignancy, immunodeficiency, or another immune dysregulating event. The diagnosis is established by meeting five of eight criteria, including fever greater than 38.5 degrees Celsius; splenomegaly; peripheral cytopenia (at least two of hemoglobin < 9 g/dL, platelets < 100, 000/microL, absolute neutrophil count < 1000/microL); hypertriglyceridemia (fasting > 265 mg/dL) or hypofibrinogenemia (<150 mg/dL); hemophagocytosis in the bone marrow, spleen, lymph node, or liver; decreased natural killer cell activity; ferritin > 500 ng/mL; and elevated soluble CD25 (IL-2 receptor *α*) two standard deviations above age-adjusted norms [2, 3]. Laboratory evaluation of soluble CD25 and natural killer cell activity require specialized testing and therefore do not result in real time. Without treatment, prognosis is limited to a few months, and the greatest barrier to survival is delay in diagnosis. Treatment entails identification and treatment of the underlying trigger. With proper treatment and identification of triggering event, survival increases to 54% at 6.2 years [4, 5]. Prognosis is worse with higher ferritin levels, underlying malignancy, older age, and low serum albumin levels [6, 7].

Although rheumatologic disease by itself can be a trigger for HLH, this is rare. Among adults on biological therapies for rheumatologic disease, infection is the most common trigger [8]. Histoplasmosis is a serious fungal infection that is endemic to the Ohio and Mississippi River valleys in the United States, as well as parts of Central and South America. The infective agent, *Histoplasma capsulatum*, is most likely to be found in soil containing bird or bat droppings. Inhalation of the spores allows them to transform into yeast and travel throughout the lymphatic and vascular system. Pulmonary histoplasmosis can cause pneumonia, mediastinal or hilar masses, pulmonary nodules, cavitary lung disease, and pericarditis. Risk factors for disseminated histoplasmosis include AIDS, immunodeficiency, immunosuppressive medications, and extremes of age. Untreated diffuse histoplasmosis is fatal within weeks, but proper antifungal treatment with amphotericin B or itraconazole increases survival rates dramatically [9].

Cases of HLH secondary to disseminated histoplasmosis are rare and extremely serious. Several case reports in the literature describe patients with AIDS [10–15], malignancy [16], or immunosuppression due to medications [17, 18]. Below we present two cases of patients with HLH secondary to disseminated histoplasmosis, both in the setting of recent immunosuppressive medication for underlying autoimmune disease.

## 2. Case Report #1

A 66-year-old woman presented to an emergency department near her home in Mexico with fever and abdominal pain of two weeks duration. She also described two months of increased fatigue and malaise, as well as increased swelling around her feet and ankles that had more recently developed. Although she was born in the United States in Oregon, she had been living in Mexico for several years. She denied travel to any other regions. A CT scan demonstrated appendicitis, and she was admitted to a hospital in Mexico for appendectomy. However, over the subsequent five days, she continued to have worsening fevers and abdominal pain. At this point, she flew to Mayo Clinic Arizona for further evaluation.

Her past medical history was significant for rheumatoid arthritis and chronic low back pain. Medications included gabapentin, daily prednisone (20 mg/day), spironolactone, and omeprazole. Following her appendix surgery, she had been placed on moxifloxacin for five days. She had been on methotrexate for several years, but this was discontinued a few months prior due to elevation of liver enzymes.

At the time of presentation, she appeared ill, and her vitals included fluid responsive hypotension, tachypnea with respiratory rate of 22, and mild hypoxia with oxygen saturation 88-95% on room air. Physical exam was significant for bibasilar crackles, abdominal distension, and diffuse tenderness to palpation without peritoneal signs, diffusely tender joints, and erythema over the left elbow and the right knee that was warm to touch. Initial laboratory evaluation demonstrated a hemoglobin of 12.5 g/dL (reference range 12.0–15.5 g/dL), platelet count of 8 × 10^(9)^/L (reference range 149–375 × 10^(9)^/L), and white blood cell count (WBC) 2.7 × 10^(9)^/L (reference range 3.4–10.6 × 10^(9)^/L) with neutrophilic predominance (absolute neutrophil count (ANC), 2.05 × 10^(9)^/L (reference range 1.40–6.60 × 10^(9)^/L)). Electrolyte panel showed hyponatremia 131 mmol/L (reference range 135–145 mmol/L) but was otherwise normal, including creatinine 0.9 mg/dL (reference range 0.6–1.1 mg/dL). LFTs were elevated with alkaline phosphatase 318 U/L (reference range 55–142 U/L), alanine aminotransferase (ALT) 145 U/L (reference range 7–45 U/L), and aspartate aminotransferase (AST) 252 U/L (reference range 9–43 U/L). Most notably, her ferritin was drastically elevated at 27,300 mcg/L (reference range 11–307 mcg/L). Additionally, triglycerides were elevated at 272 mg/dL (reference range < 150 mg/dL), and fibrinogen was reduced at 120 mg/dL (reference range 200–430 mg/dL). Chest X-ray showed trace bilateral pleural effusions and small calcified granulomata in the upper lungs bilaterally. Given her diffusely tender joints, X-ray of the hands were performed and revealed numerous subchondral cysts and erosion, as well as subluxation of the right index finger distal phalanx, with sparing of the metacarpophalangeal joints consistent with rheumatoid arthritis. Due to tachypnea, leukopenia, and hemodynamic instability, she was diagnosed with sepsis.

**Figure 1:**
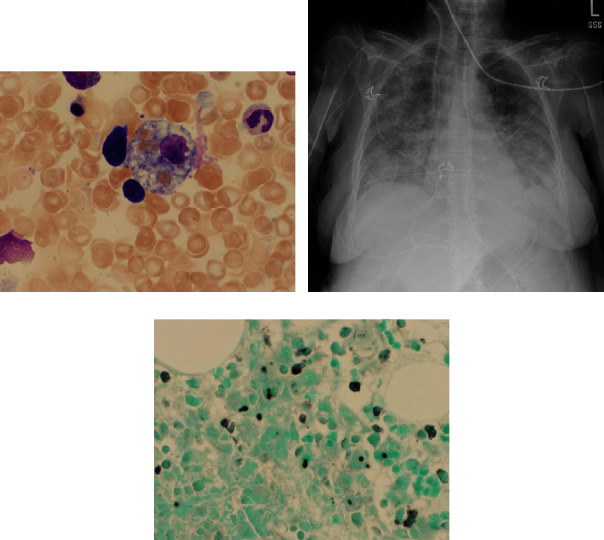
(a) Bone marrow aspirate reveals evidence of hemophagocytosis. (b) Chest X-ray reveals evidence of bilateral infiltrates, and patient then diagnosed with ARDS. (c) Bone marrow core biopsy GMS stain reveals histoplasmosis.

She initially received broad spectrum antibiotics and underwent paracentesis which was negative for peritonitis. Bacterial and fungal cultures of the ascitic fluid showed no growth. Her significantly elevated ferritin prompted a bone marrow biopsy (BMB), which showed features of hemophagocytic lymphohistiocytosis (Figure 1(a)). Given her findings of pancytopenia, elevated ferritin and triglycerides, reduced fibrinogen, and evidence of hemophagocytosis on BMB, she was treated with high-dose steroids and intravenous fluids. After initiation of steroids, her thrombocytopenia and joint pain improved. However, her WBC count continued to drop, and the patient continued to spike fevers; thus, antibiotics were adjusted to cover for neutropenic fever. Without an identified infectious source, a CT of the brain, chest, abdomen, and pelvis was performed which showed bilateral pulmonary infiltrates but was otherwise unremarkable. Liver biopsy was considered but was deemed unsafe due to coagulopathy. Her respiratory status declined eventually requiring high-flow oxygen due to acute respiratory distress syndrome (ARDS) (Figure 1(b)), and she was treated with aggressive diuresis. Initially, she had adequate response but ultimately developed oliguria and renal failure.

On hospital day four, the ferritin had improved to 609 mcg/L (reference range 11–307 mcg/L); however, LFTs continued to rise, and resultant coagulopathy required daily cryoprecipitate transfusion to maintain safe fibrinogen levels. Due to worsening clinical status with multiorgan failure including the liver, kidney, respiratory, and bone marrow, it was decided to add etoposide for more aggressive treatment of HLH. Despite these efforts, her ferritin rapidly climbed to over 80,000 mcg/L (reference range 11–307 mcg/L). Infectious workup to date was negative including blood bacterial and fungal cultures, ascites bacterial and fungal cultures, and blood tests for HIV, HHV-6, EBV DNA, hepatitis A and E IgM, parvovirus B19, adenovirus, and tuberculosis quantiferon.

Infectious etiology was identified when her urine histoplasmosis antigen returned positive above the upper detectable limit. The bone marrow biopsy was stained for histoplasmosis and confirmed disseminated infection (Figure 1(c)). Amphotericin therapy was initiated as the patient began CRRT for worsening kidney failure. Over the next eight days, however, she did not show any clinical improvement and was treated supportively for ICU delirium, respiratory failure, anuria requiring intermittent hemodialysis, worsening coagulopathy and rising ammonia, and malnutrition requiring tube feeds. On day twelve, voriconazole was added for additional antifungal coverage.

Unfortunately, despite these efforts, the patient's blood counts continued to decline, and she developed massive melena on hospital day 13 with a hemoglobin of 4 g/dL (reference range 12.0–15.5 g/dL), WBC 0.1/L (reference range 3.4–10.6 × 10^(9)^/L), ANC 0.11/L (reference range 1.40–6.60 × 10^(9)^/L), and platelets 41/L (reference range 149–375 × 10^(9)^/L). At this point, the patient and her power of attorney elected to withdraw aggressive treatments, and she was transferred to inpatient hospice.

## 3. Case Report #2

**Figure 2:**
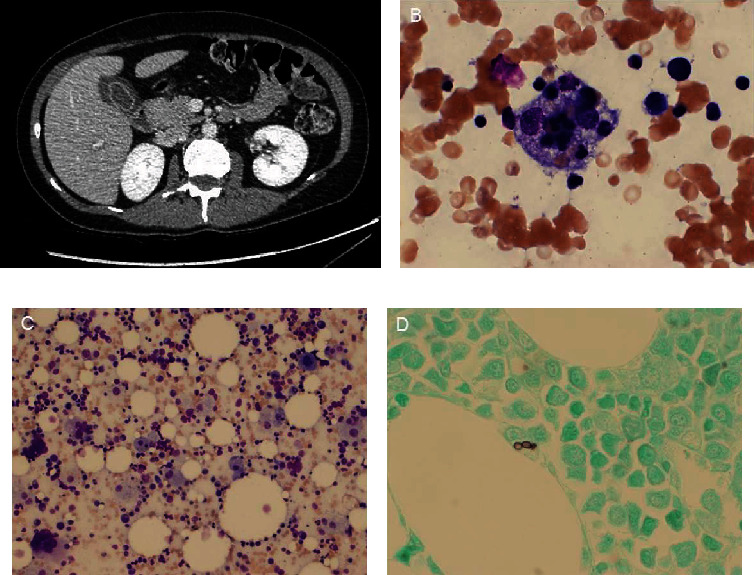
(a) CT abdomen on admission reveals edematous gallbladder which was later found to have evidence of *Histoplasma capsulatum*. (b, c) Bone marrow aspirate reveals hemophagocytosis. (d) GMS stain of bone marrow core biopsy positive for histoplasmosis.

A 41-year-old woman residing in Arizona with a history of ulcerative colitis from who was on Remicade up until one month earlier was admitted three times over a course of a month with a variety of symptoms without identifying etiology. Initially, she had nonspecific fevers, headache, neck pain, fatigue, nausea, vomiting, and arthralgias. She was found to have an edematous gallbladder (Figure 2(a)) and mild splenomegaly, with a negative infectious workup. With symptomatic care, her fevers were controlled, and she was discharged with a diagnosis of presumed viral syndrome versus Remicade toxicity. However, symptoms worsened, and she represented with fever, sepsis, cholestatic jaundice, acute kidney injury, pancytopenia, and multiple metabolic abnormalities requiring admission to the intensive care unit. Initially, she underwent ERCP with sphincterotomy, and common bile duct stent was placed. On endoscopy, the duodenal mucosa was noted to be friable, biopsy revealed reactive gastropathy, and *H. pylori* testing was negative. She then underwent laparoscopic cholecystectomy for acalculous cholecystitis. Postoperatively, she developed bloody secretions from the endotracheal tube followed by ARDS; thus, she was unable to be extubated. She also had episodes of epistaxis and melena through this time. Her clinical status quickly deteriorated to multiorgan failure including renal failure requiring dialysis, cholestatic liver failure, respiratory failure requiring mechanical ventilation, and critical pancytopenia requiring high-volume transfusions including 17 units of packed red blood cells, 17 units of platelets, and 6 units of cryoprecipitate.

Workup through this point revealed a markedly elevated ferritin of 73,469 mcg/L (reference range 11–307 mcg/L), elevated lactate dehydrogenase of 2,375 U/L (reference range 122–222 U/L), hypofibrinogenemia of 135 mg/dL (reference range 200-430 mg/dL), and hypertriglyceridemia 2,259 mg/dL (reference range < 150 mg/dL), raising suspicion for HLH. Bone marrow biopsy confirmed the diagnosis (Figures 2(b)–2(d)), and she was initiated on high-dose steroids and etoposide. She developed a severe diffuse rash to etoposide necessitating its discontinuation. Investigation for an underlying trigger included bronchoalveolar lavage (BAL) culture which returned positive for *Histoplasma capsulatum*, in addition to positive histoplasma urinary antigen. During the same hospitalization, she additionally underwent cholecystectomy, and cultured gallbladder tissue ultimately grew *H. capsulatum*. Based on history, this was likely acquired in the setting of prior Remicade use while exploring bat caves in Cancun, Mexico, two weeks prior to admission. The patient was born and lived in Arizona until her presentation and denied travel to any endemic areas. Her Aspergillus antigen was also positive from BAL, but this was felt to be a false positive as Aspergillus antigen testing cross-reacts with Histoplasma.

For treatment of disseminated histoplasmosis, she received intravenous AmBisome starting on day 4 of hospitalization and later transitioned to oral itraconazole. With these treatments, she recovered well and was eventually transferred out of the intensive care unit on day 18. She required one dose of Neupogen for leukopenia, but her white blood cell count recovered thereafter, and platelet count returned to normal. Her ferritin had dramatically decreased to 1,568 mcg/L (reference range 11–307 mcg/L). Her kidney and liver function recovered to the point where she could be safely discharged to acute rehabilitation after an approximately four weeks stay in the hospital. She continued to improve during a two-week rehabilitation stay and was then discharged home with an oral steroid taper and oral itraconazole for one year following diagnosis.

## 4. Discussion

The diagnosis and treatment of acquired HLH in adults are serious and challenging, as the clinical presentation can be fairly nonspecific and diagnosis is based on a collection of both clinical and laboratory abnormalities. In pediatric patients with familial HLH, an elevated ferritin greater than 10,000 *μ*g/L is highly sensitive and specific for diagnosis [19]. In adults, however, elevated ferritin has been linked to various conditions including malignancy, liver dysfunction, infection, anemia of chronic disease, or iron overload [20]. Markedly elevated ferritin, however, is thought to be more specific for adult onset stills disease, macrophage activation syndrome, and HLH. In a retrospective study [20] of patients with ferritin levels greater than 50,000 *μ*g/L, 19% were found to have HLH. The most frequently seen diagnosis in these patients was renal failure (73%), liver injury (61%), infections (52%), and hematologic malignancies (36%). No ferritin cut off level was found to be specific for HLH in this study. Currently, ferritin > 500 *μ*g/L is included in the diagnostic criteria for HLH, although many prefer to use >3000 *μ*g/L. Given the lack of specific criteria for diagnosis of adult acquired HLH, a scoring system which includes the above mentioned diagnostic criteria for HLH was developed and has been shown to be 90% sensitive and 79% specific, although specificity decreases as patients become less stable [21]. Once the diagnosis is confirmed, treatment must be initiated immediately. Additionally, a search must be undertaken to find an underlying trigger for HLH; as without treating the source, there is little chance for clinical improvement.

Here, we present two cases of HLH secondary to disseminated histoplasmosis. Previously, published cases of histoplasmosis associated HLH were reported in HIV [22], hematologic malignancy [16], and transplant patients on immunosuppression [18, 22]. Disseminated histoplasmosis by itself is also quite serious but generally treatable with antifungal therapy. Current IDSA guidelines for moderate to severe disease include liposomal amphotericin B (3.0 mg/kg daily) for 1-2 weeks, followed with oral itraconazole for at least 12 months [9]. Patients who have both disseminated histoplasmosis and HLH portend a worse prognosis. In a retrospective review of eleven cases of histoplasmosis associated HLH, 45% died within 30 days, and 63% died within 90 days [22]. The main challenge of caring for patients with histoplasmosis associated HLH is management of the multiorgan failure that can result from this combination of diseases. Both HLH and histoplasmosis can cause hematologic abnormalities, namely, pancytopenia, and both patients presented here required large volume transfusions. In addition, both patients developed respiratory failure from ARDS, renal failure requiring renal replacement therapy, liver failure, and gastrointestinal bleeding. Disseminated histoplasmosis involving the gastrointestinal system can cause cholecystitis and appendicitis leading to surgery, as well as melena and gastritis, which were seen in these cases.

The main risk factors for disseminated histoplasmosis leading to HLH in these cases were immunosuppressive medication (methotrexate and infliximab) and environmental exposure (bird or bat droppings, especially in caves). Interestingly, in both cases, the offending medication had been discontinued due to laboratory abnormalities 1-2 months prior to any presenting symptoms. It is unclear whether this may have been a risk factor for disease. Nevertheless, with a presentation of diffuse infectious symptoms without an identified etiology in the context of recent immunosuppressive medication, an exhaustive history and laboratory inquiry should be pursued to avoid delayed diagnosis for serious conditions such as disseminated fungal infection and HLH.

## Data Availability

No datasets were used.
